# Improvement of *Eucalyptus* sp for biomass and bioenergy production in the north of Spain

**DOI:** 10.1186/1753-6561-5-S7-P43

**Published:** 2011-09-13

**Authors:** Carmen Diaz-Sala, Maria Dolores Velez, Elena Carneros, Dolores Abarca, Carmen Barbero, Alicia Del Amo, Jose Antonio Cabezas, Claudia Escudero, Libertad Juez, Carlos Tejedor, Maria Teresa Cervera

**Affiliations:** 1 Department of Plant Biology, University of Alcalá, 28871 Alcalá de Henares, Madrid, Spain; 2 Departamento de Ecología y Genética Forestal INIA-CIFOR, Ctra. de La Coruña Km 7,5, 28040 Madrid, Spain; 3 Departamento de Investigación Agroalimentaria, Instituto Madrileño de Investigación y Desarrollo Rural, Agrario y Alimentario, Finca El Encín. Ctra A-2, Km38200, 28800 Alcalá de Henares, Spain; 4 SNIACE. Ctra. Ganzo, s/n, 39300 Torrelavega, Cantabria, Spain

## 

*Eucalyptus globulus Labill.* has been used in Spain for decades as a cellulose source for the paper and textile industry. Since 1997, Sniace group has tested new provenances and families from 29 different *Eucalyptus* species to explore their capacity for biomass and bioenergy production.

Plus trees for growth, wood quality, rooting capacity and tolerance to *Mycosphaerella sp* have been identified by mass selection, and more than 300 controlled crosses among those trees have been carried out. However, the restrictions caused by the high susceptibility to *Mycosphaerella* leaf disease and the low rooting capacity of the species *Eucalyptus globulus Labill.* delay the application of the gains to a commercial scale.

The objective of this project is to improve the production of *Eucalyptus* in the North of Spain focused on the improvement of two traits of economic importance: clonal/rooting capacity and tolerance to *Mycosphaerella sp.* To achieve this goal three partial objectives have been approached:

## Mass propagation of selected adult clones and rooting improvement by in vitro tissue culture

Clonal propagation by sequential subcultures of axillary buds in proliferation and elongation media and further rooting of elongated shoots will be developed. This will further optimize a simple *in vitro* protocol for the micropropagation of identified elite mature trees for raising plantations. Micropropagation can maintain selection gains, developed in improvement programmes, to be transmitted directly to plantations or to seed orchards, adding value to the subsequent products and reducing production costs in the long term. For this purpose, it is required to test differences between in vitro produced trees and seed-derived trees, regarding some important traits such as juvenility, growth, productivity, uniformity and morphological traits.

## Certification of clones and varieties by the use of specific molecular markers

A collection of 97 elite clones have been genotyped using 22 SSRs [1,2], and four multiplex PCR reactions. This analysis has allowed the genetic characterization of each individual clone, the analysis of genetic similarity and inferring tentative relatedness among clones. This information will be used to improve crossing designs as well as to certify clonal material.

## Genetic dissection of rooting capacity and tolerance to Mycosphaerella sp

A strategy combining transcriptomics, genetic mapping, phenotypic characterization of the targeted traits and QTL analysis has been established. For this purpose a mapping progeny was obtained using two contrasting progenitors: A tree with high rooting capacity, tolerant to *Mycosphaerella* sp, and a tree with low rooting capacity, susceptible to *Mycosphaerella* sp. The steps to reach this goal include: 1) Construction of a cDNA library made of pooled RNAs, from different tissues collected from the progeny plants grown under different conditions. This cDNA library is used as template for Roche GS-FLX Titanium high throughput sequencing. Once the unigene is identified, SNPs from selected genes are chosen to design a 1536 Golden Gate array which is used to genotype both progeny individuals and progenitors. Segregating SNPs are used to construct genetic maps. 2) Evaluation of rooting capacity and response to infection during two consecutive years. 3) Identification of association between SNPs and trait parameters using QTL analysis followed by identification of favourable alleles (SNP variant).
